# TGFβ signaling in the brain increases with aging and signals to astrocytes and innate immune cells in the weeks after stroke

**DOI:** 10.1186/1742-2094-7-62

**Published:** 2010-10-11

**Authors:** Kristian P Doyle, Egle Cekanaviciute, Lauren E Mamer, Marion S Buckwalter

**Affiliations:** 1 Departments of Neurology and Neurological Sciences, and Neurosurgery, Stanford University Medical School, Stanford, CA, 94305-5489, USA

## Abstract

**Background:**

TGFβ is both neuroprotective and a key immune system modulator and is likely to be an important target for future stroke therapy. The precise function of increased TGF-β1 after stroke is unknown and its pleiotropic nature means that it may convey a neuroprotective signal, orchestrate glial scarring or function as an important immune system regulator. We therefore investigated the time course and cell-specificity of TGFβ signaling after stroke, and whether its signaling pattern is altered by gender and aging.

**Methods:**

We performed distal middle cerebral artery occlusion strokes on 5 and 18 month old TGFβ reporter mice to get a readout of TGFβ responses after stroke in real time. To determine which cell type is the source of increased TGFβ production after stroke, brain sections were stained with an anti-TGFβ antibody, colocalized with markers for reactive astrocytes, neurons, and activated microglia. To determine which cells are responding to TGFβ after stroke, brain sections were double-labelled with anti-pSmad2, a marker of TGFβ signaling, and markers of neurons, oligodendrocytes, endothelial cells, astrocytes and microglia.

**Results:**

TGFβ signaling increased 2 fold after stroke, beginning on day 1 and peaking on day 7. This pattern of increase was preserved in old animals and absolute TGFβ signaling in the brain increased with age. Activated microglia and macrophages were the predominant source of increased TGFβ after stroke and astrocytes and activated microglia and macrophages demonstrated dramatic upregulation of TGFβ signaling after stroke. TGFβ signaling in neurons and oligodendrocytes did not undergo marked changes.

**Conclusions:**

We found that TGFβ signaling increases with age and that astrocytes and activated microglia and macrophages are the main cell types that undergo increased TGFβ signaling in response to post-stroke increases in TGFβ. Therefore increased TGFβ after stroke likely regulates glial scar formation and the immune response to stroke.

## Background

Transforming Growth Factor β1 (TGF-β1) is universally induced by acute and chronic brain injury, including stroke, trauma, seizure, multiple sclerosis, and Alzheimer's disease [1]. TGFβ is highly conserved and in mammals exists as three isoforms that bind to the same receptors; TGF-β1, TGF-β2 and TGF-β3. TGF-β1 is the isoform typically induced by injury [2]. In the brain, TGFβ receptors are present on all major cell types [3]. However, both TGFβ activation and signaling are extensively regulated and due to this its effects are typically pleiotrophic and context-dependent. Therefore, after brain injury the biological effects of TGFβ signaling are likely influenced by both injury type and timing.

After brain injury, TGFβ signaling can be neuroprotective, but also promote glial scarring and fibrosis [1,4-6]. While neuroprotection is undoubtedly desirable, glial scarring and fibrosis are less likely to be advantageous. In addition, TGFβ's have potent effects on the immune system. TGF-β1 can be stimulatory or inhibitory depending on the cell type, cytokine milieu and differentiation state of the responding cell and can have both pro- and anti-inflammatory effects [7]. It is not known which subset of these roles TGF-β1 plays when it is induced by brain injury and how this varies in different types of brain injury.

In stroke, TGF-β1 mRNA is elevated for at least a week afterwards [8] and clearly exerts a neuroprotective role. When overexpressed, TGF-β1 limits stroke size [4,9,10]. And when TGFβ signaling is blocked, ischemic damage is exacerbated [11]. Because of this, it may be an effective therapeutic agent for stroke. Developing new therapeutic agents to treat those affected by stroke is critical. The elderly segment of the American population is rapidly increasing and 70% of the deaths of individuals over 65 are attributable to cardiovascular disease [12]. But in order to develop TGFβ as a therapeutic agent, it is necessary to understand if it plays any additional roles in the brain after stroke.

We therefore investigated the timecourse and cell-specificity of TGFβ signaling after stroke, and whether its signaling pattern is altered by gender and aging in a mouse model of stroke. We report here that TGFβ signaling increases 2 fold after the dMCAO model of stroke, beginning on day 1 and peaking on day 7. This pattern of increase is similar in each gender and preserved in old animals, although absolute TGFβ signaling was much higher in the brains of the aged animals. CD68+ activated microglia and macrophages were the predominant source of increased TGF-β1 after stroke and astrocytes and CD68+ cells were the main cell types that responded to the post-stroke increase in TGF-β1. Our results demonstrate that TGFβ signaling peaks at 7 days after dMCAO stroke, increases with aging, and that the cells with increased responses during this time period, astrocytes and microglia, are those involved in glial scarring and innate immune responses. This may indicate that TGFβ signaling plays a role in regulating both the immune response and the glial scar after stroke.

## Methods

### Animals

SBE-LucRT mice were a gift from Dr Tony Wyss-Coray [13]. Nine females and 5 males (5 months old) were used to gauge variability in bioluminescence between individual mice, and to determine if bioluminescence is affected by the estrus cycle. Eighteen young females (5 months old) and eighteen old females (18 months old) were used to measure TGFβ signaling after stroke in real time by bioluminescence. 3 mice from each cohort were sacrificed at days 1, 3, 7, 14 and 21 after stroke for localization of TGF-β1 and TGFβ signaling by immunoflorescence. Three mice of each age group also underwent sham surgery and were imaged for 21 days prior to sacrifice for localization of TGFβ signaling in uninjured brains. This experimental paradigm was repeated in young and old male mice.

### Surgery

Distal middle cerebral artery occlusion (dMCAO) was induced as described previously [14]. Briefly, mice were anesthetized by isoflurane inhalation (2% isoflurane in 100% oxygen) and the skin over the right temple was shaved. Skin was swabbed with chlorehexidine and an incision was made to expose the temporalis muscle. A pocket was created in the temporalis muscle to expose the skull underneath and the right middle cerebral artery (MCA) was identified. A microdrill was used to penetrate the skull and expose the underlying MCA. The meninges were cut and the vessel was ligated using a small vessel cauterizer. The temporalis muscle was replaced and the wound closed using surgical glue. Throughout surgery body temperature was maintained at 37°C using a feedback controlled heating blanket.

### Bioluminescent imaging

Bioluminescence was detected as described previously using the In Vivo Imaging System (IVIS; Xenogen, Alameda, CA) [13]. Mice were injected intraperitoneally with 150 mg/kg D-luciferin (Xenogen) 10 min before imaging and anesthetized with isoflurane during imaging. Photons emitted from living mice were acquired as photons per second per cm^2 ^per steradian (sr) by using LIVINGIMAGE software (Xenogen) and integrated over 5 min. For photon quantification, a region of interest was manually selected and kept constant within all experiments. Baseline imaging was performed 1 week before stroke was induced and bioluminescence was expressed as fold induction over baseline levels.

### Estrus cycle determination

For determination of stage of estrus, sterile cotton swabs moistened with saline were inserted into the vagina and gently rotated. Swabs were then rolled onto glass slides and allowed to dry. Polymorphonuclear lymphocytes (PMNs) and epithelial cells were visualized with a Hema 3 Stat Pack (Fisher Cat#23-123-869) used according to the manufactuer's directions. Stage of estrus cycle was determined based on the ratio of PMN's to nucleated and cornified epithlieal cells. Diestrus was defined as the stage when cells consisted mainly of polymorphonuclear lymphocytes (PMNs). Proestrus was defined as the stage when nucleated and cornified epithelial cells were observed, along with a few PMNs. Estrus was classified as the stage when cornified epithelial cells were predominant, and metestrus was defined as the stage when cornified epithelial cells and PMNs were detected.

### Infarct evaluation

To visualize the region of infarction, representative animals from each age group (n ≥ 5) were sacrificed at 24 hours post stroke and their brains processed for TTC staining. Brains were sectioned into 1 mm thick slices and each slice was immersed in 1.5% TTC in PBS at 37°C for 15 minutes and then fixed in 10% formalin. The area of the ipsilateral hemisphere and the area of the infarct on each section was measured in a blinded fashion using NIH ImageJ 1.43u. The measurements were multiplied by the distance between sections (1 mm) and then summed over the entire brain to yield volume measurements.

### Immunoflorescence

Immunoflorescence was performed on PFA fixed free-floating coronal brain sections (40 μm) using standard techniques. The following primary antibodies were used: anti-TGF-β1 (1:1000; Torrey Pines Biolabs, East Orange, NJ), anti-pSmad2 (1:1000; Cat# AB3849, Millipore, Billerica, MA); Milli-Mark Pan Neuronal marker (1:500; Millipore, Billerica, MA); anti-GFAP (1:1000; Dako, Carpinteria, CA); anti-CD68 (1:1000; ABD Serotec, Raleigh, NC); anti CAII (1:2000; ABD Serotec, Raleigh, NC) and anti-β-dystroglycan (1:1000; Novacastra/Leica Microsystems, Bannockburn, IL). For quantification of Milli-Mark Pan Neuronal marker, CAII, CD68 and GFAP colocalization with pSmad2, two sections from each mouse spaced 300 μm apart were immunostained. From each section four random fields of view in the penumbra (40× objective) were analyzed. The total number of cells positive for each cell marker was averaged and the percentage of each population that was also positive for pSmad2 was calculated.

### Western blotting

Unstroked and stroked brains were lysed in glo lysis buffer (Promega). Protein concentrations were equalized and lysates mixed with 4× NuPage LDS loading buffer (Invitrogen). Samples were loaded onto 4-12% NuPage Bis-Tris gels (Invitrogen) and subsequently transferred onto PVDF membranes. Blots were incubated with rabbit polyclonal antibodies (1:1000) against pSmad2 (cat# AB3849 from Millipore and cat# 3101 from Cell Signaling) and actin (cat# A5060 Sigma-Aldritch) and horseradish peroxidase-conjugated goat anti-rabbit IgG as secondary antibody. Protein signals were detected using an ECL kit (Amersham Pharmacia Biotech) and band intensities were quantified on the original digital image using UN-Scan-It Gel 6.1.

### Statistical analysis

Data was acquired in a blinded and unbiased fashion. Data are expressed as mean +/- standard error of the mean (SEM). Statistical analyses were performed with Prism 5 software (GraphPad, San Diego, CA). Means between two groups were compared with two-tailed, unpaired Student's t tests; comparisons of means from multiple groups with one control were analyzed with one-way ANOVA and Dunnet's post hoc test.

## Results

### TGFβ signaling in the brain is equivalent in males and females and does not fluctuate with the estrus cycle

**Figure 1:**
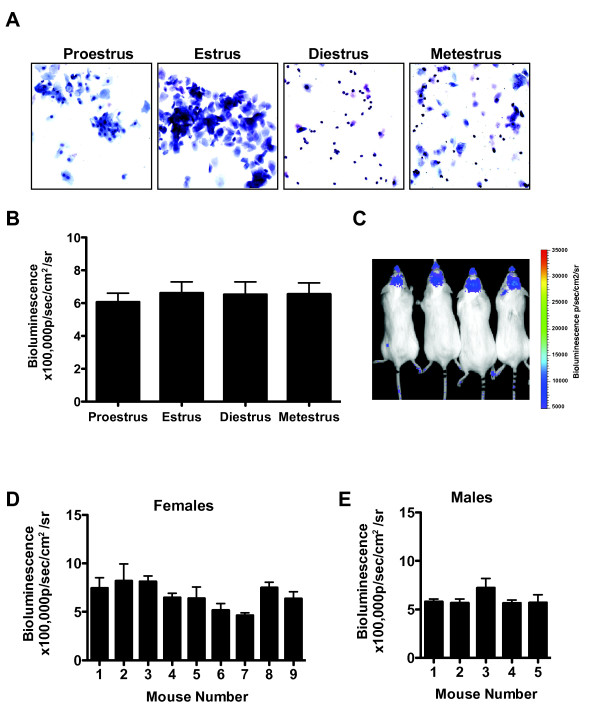
**TGF-β signaling in the brain does not fluctuate with the estrus cycle or gender and is equivalent between individuals**. A.) Images depicting each stage of the estrus cycle. Vaginal swabs were stained by a Hema 3 Stat Pack. B.) Average bioluminescence for each day of the estrus cycle (n = 9). C.) Representative bioluminescence showing the consistency of reporter gene (luciferase) expression (4 females shown). D.) Average bioluminescence from 9 females imaged for 10 days. There is no significant difference between individuals. E.) Average bioluminescence from 5 males imaged for 10 days. There is no significant difference between individuals. Error bars, SEM.

TGFβ pathway activation is transmitted primarily through the Smad system. Receptor-regulated Smads (Smad 1, 2, 3, 5 or 8) are phosphorylated, associate with a co-Smad (Smad 4) and translocate to the nucleus, where they bind to Smad binding elements (SBE) to activate transcription of target genes [15,16]. SBE-LucRT mice are TGFβ reporter mice that express a fusion protein consisting of luciferase (luc), red fluorescent protein (R) and thymidine kinase (T) under the control of a SBE [13]. In these mice luciferase bioluminescence is predominantly in the brain, reliably indicates TGFβ signaling, and can be followed in real time [13,17]. To determine how comparable reporter gene expression is between individual SBE-LucRT mice, and if reporter gene expression fluctuates with gender and the estrus cycle, we imaged nine female and 5 male SBE-LucRT mice for ten days. To determine which stage of the estrus cycle each female was in, a vaginal smear was taken every day and stained with a modified Wright-Giemsa stain. The number of polymorphonuclear leukocytes (PMNs) present in each smear was compared to the number of nucleated and cornifed epithelial cells (Fig 1A). There was no significant difference in bioluminescence between each female or for each stage of the estrus cycle (Fig 1B-D). We also found reporter gene expression to be comparable between male mice and equivalent to that of the females (Fig 1E). This indicates that bioluminescence in SBE-LucRT mice is very comparable between individuals and that TGFβ signaling in the brain does not fluctuate with gender or the estrus cycle.

### TGFβ signaling increases with age and stroke

**Figure 2:**
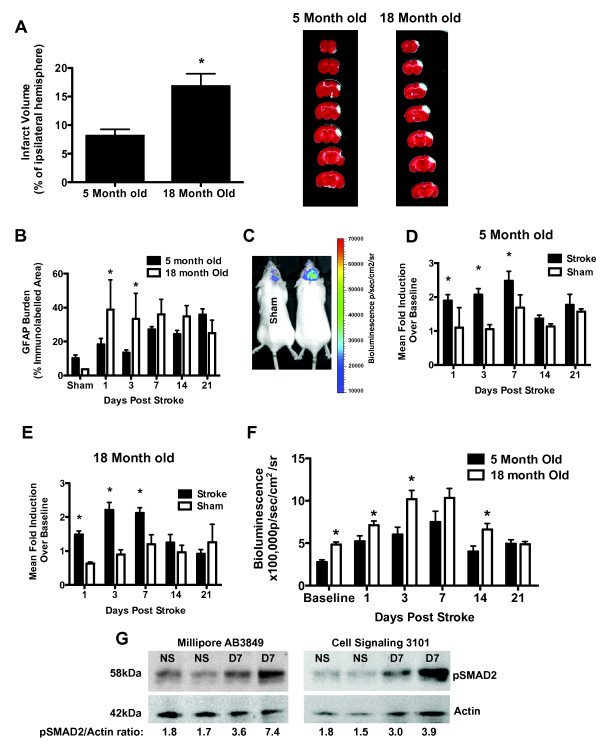
**TGFβ signaling in the brain increases with age and stroke**. A.) Infarct volume and representative TTC images of the lesion 24 hours following dCMAO (*p < 0.05). B.) Quantification of GFAP immunostain, showing that astrogliosis is accelerated in aged mice (*p < 0.05 compared to 5 month old). C.) Representative bioluminescence showing reporter gene (luciferase) expression is increased after stroke compared to surgical shams (2 females shown at 3 days post stroke). D.) Time course of TGFβ signaling (luciferase induction) after stroke in 5 month old females (*p < 0.05 compared to sham). E.) Time course of TGFβ signaling (luciferase induction) after stroke in 18 month old females (*p < 0.05 compared to sham). F.) Comparison of absolute values of bioluminescence between young and old mice, showing that TGFβ signaling in the brain increases at baseline and after stroke (*p < 0.05 compared to 5 month old). G.) Western blot to corroborate that TGFβ signalling increases after stroke. Two different antibodies for pSmad2 were used to confirm that pSmad2 is increased at day 7 after stroke. NS = no stroke, D7 = day 7 post stroke. All error bars for this figure are SEM.

The dMCAO model of stroke produces a small lesion in the cortex with a clearly defined border and a small penumbra. In line with previous reports infarct volume was significantly larger in the aged mice and astrogliosis (GFAP immunoreactivity) was accelerated (Fig 2A, B) [18,19]. Bioluminescence in the 5 month old females was increased 2 fold on day 1 post stroke, remained elevated for 7 days post stroke and was localized to the region of the infarct (Fig 2C). After 7 days TGFβ signaling began to return to baseline (Fig 2D). This fold increase was similar in the 18 month old female mice (Fig 2E), although the absolute amount of TGFβ signaling in the old mice was substantially higher (Fig 2F). This is not surprising considering that the lesion was much larger in the aged animals. However, TGFβ signaling was also significantly higher in the old animals at baseline, demonstrating that in the absence of injury TGFβ signaling in the brain increases with age.

To confirm the increase in TGFβ signaling we detected by luciferase activity, we performed a western blot on brain extracts from the ipsilateral hemisphere of mice sacrificed before, and 7 days after stroke, for phosphorylated Smad2 (pSmad2), a downstream mediator of TGFβ signaling. The pSmad2 antibody we used for immunoflorescence (Millipore AB3849) detected a protein with the correct molecular weight for pSmad2 (58 kDa) and bound the same band as an alternative pSmad2 antibody (Cell Signaling 3101; Fig 2G). This verifies that the pSmad2 antibody we used for subsequent immunoflorescence is specific for pSmad2. In accordance with our luciferase findings pSmad2 levels were increased 7 days post stroke (Fig 2G).

In summary, TGFβ signaling increases 2 fold after dMCAO stroke, beginning on day 1 and peaking on day 7, this pattern of increase is preserved in old animals, and absolute TGFβ signaling in the non-injured brain increases with age. We did not observe a gender difference in these findings or in infarct volume.

### TGF-β1 is predominantly co-localized with CD68+ cells after stroke

**Figure 3:**
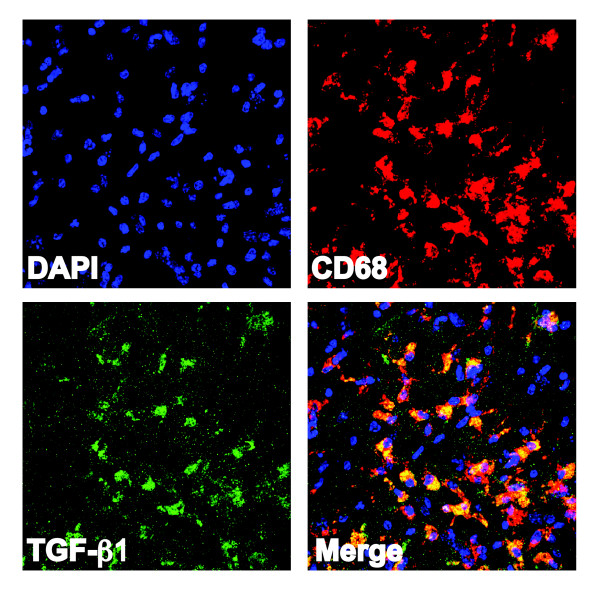
**TGF-β1 is predominantly colocalized with CD68+ cells after stroke**. Representative image (40×) showing CD68 (activated microglia/macrophages) co-localized with TGF-β1 after stroke (images are from a young female mouse at day 3 post stroke.

To determine which cell type is the likely source of increased TGF-β1 production after stroke, brain sections were stained with an anti-TGF-β1 antibody, colocalized with markers for astrocytes (GFAP), neurons (Milli-mark Pan-Neuronal marker), and monocytic immune cells (CD68). Although TGF-β1 colocalized with all three cell types it was predominantly co-localized with CD68, which marks both activated microglia and macrophages. These cells were located in both the ischemic penumbra and the stroke core. These data suggest that endogenous microglia and invading macrophages are the predominant cell types that make TGF-β1 after ischemic brain injury (Fig 3).

### TGFβ signaling in neurons, oligodendrocytes and endothelial cells is unchanged by stroke

**Figure 4:**
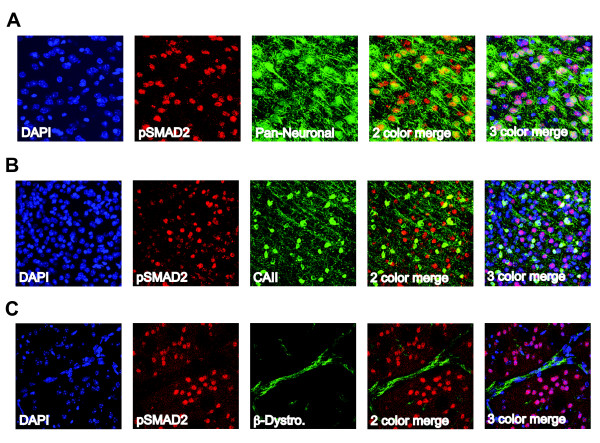
**TGFβ signaling in neurons, oligodendrocytes and endothelial cells is unchanged by stroke**. A.) Co-localization of pSmad2 (marker of TGFβ signaling) with Millimark Pan-Neuronal antibody cocktail (marker of neurons) See additional file 1 for a 3D projection. B.) Co-localization of pSmad2 with CAII (oligodendrocyte marker) See additional file 2 for a 3D projection. C.) Co-localization of pSmad2 with β-dystroglycan (endothelial cell marker) See additional file 3 for a 3D projection. Images (40×) are from young females at day 3 post stroke but are representative of all time points for both young and old, male and female mice.

**Figure 5:**
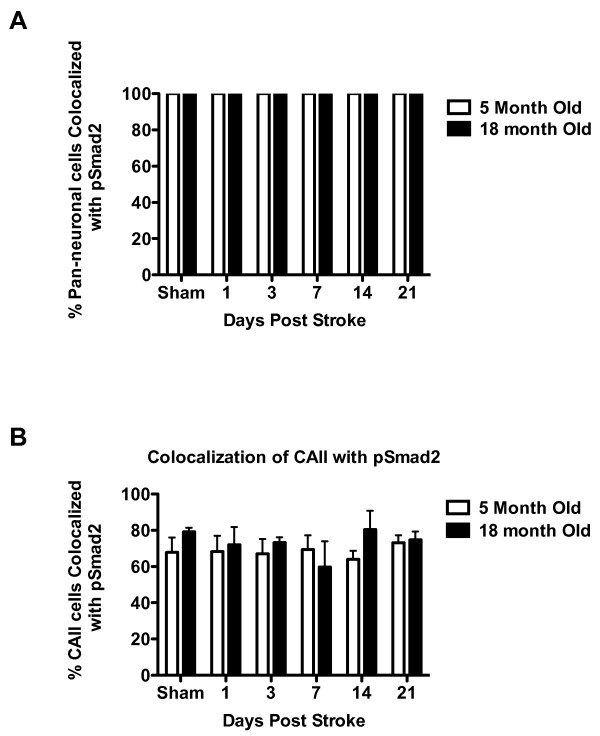
**Co-localization of pSmad2 with neurons and oligodendrocytes after stroke**. A.) Both before and after stroke 100% of neurons in the cortex are co-localized with pSmad2 in young and old mice. B.) Both before and after stroke ~70% of oligodendrocytes in the cortex are co-localized with pSmad2 in young and old mice.

To determine which cells are responding to TGFβ in the uninjured brain and after stroke, brain sections from mice sacrificed at days 1, 3, 7, 14 and 21 after stroke and at 21 days after sham surgery were double-labeled with anti-pSmad2, a marker of TGFβ signaling, and markers of neurons (Millimark Pan Neuronal marker), oligodendrocytes (CAII), endothelial cells (β-dystroglycan), astrocytes (GFAP) and activated microglia and macrophages (CD68). We found that neurons uniformly respond to TGFβ in the absence of injury and at all time points after stroke, with 100% of neurons co-localizing with pSmad2 (Fig 4A and Fig 5A; see additional file 1 to confirm colocalization by 3D reconstruction). We also found that the majority (approximately 70%) of oligodendrocytes consistently respond to TGFβ in the absence of injury and did not observe any differences after stroke (Fig 4B and Fig 5B; see additional file 2 to confirm colocalization by 3D reconstruction). There was no qualitative difference in the intensity of colocalization of pSmad2 with neurons or oliogdendrocytes, at any time point after stroke. This data suggests that the response of neurons and oligodendrocytes to TGFβ is unchanged by ischemic injury. Unlike neurons and oligodendrocytes, β-dystroglycan rarely colocalized with pSmad2 in the absence of injury and after stroke, indicating that endothelial cells in adult brain seldom signal via pSmad2 in response to TGFβ (Fig 4C; see additional file 3 to confirm colocalization by 3D reconstruction). This data was consistent across age and gender.

### TGFβ signaling increases in astrocytes and microglia/macrophages after stroke

**Figure 6:**
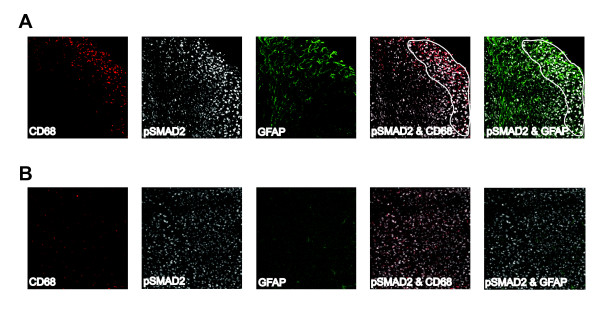
**pSmad2 immunoreactivity is increased in the stroke penumbra and colocalizes with CD68 and GFAP**. Representative images (10×) showing CD68 and GFAP colocalized with pSmad2 after stroke (image A is from a young female mouse at day 14 post stroke, image B is from a young female mouse after sham surgery). There is a qualitative increase in pSmad2 immunoflorescence in the infarcted region (outlined) with the brightest cells co-localizing with CD68 and GFAP.

**Figure 7:**
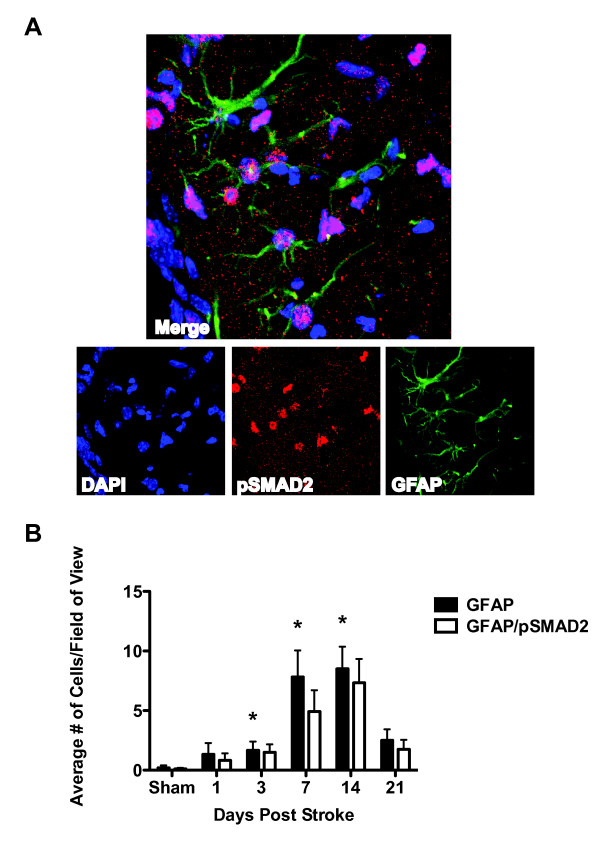
**TGFβ signaling in reactive astrocytes increases in the stroke penumbra after stroke**. A.) Representative image (40×) showing GFAP (astrocytes) colocalized with pSmad2 after stroke. Image is from a young female mouse at day 14 post stroke. See additional file 4 for a 3D projection B.) GFAP immunoreactivity increases in the first 7 days after stroke with the majority of astrocytes colocalizing with pSmad2 (*p < 0.05 compared to sham).

**Figure 8:**
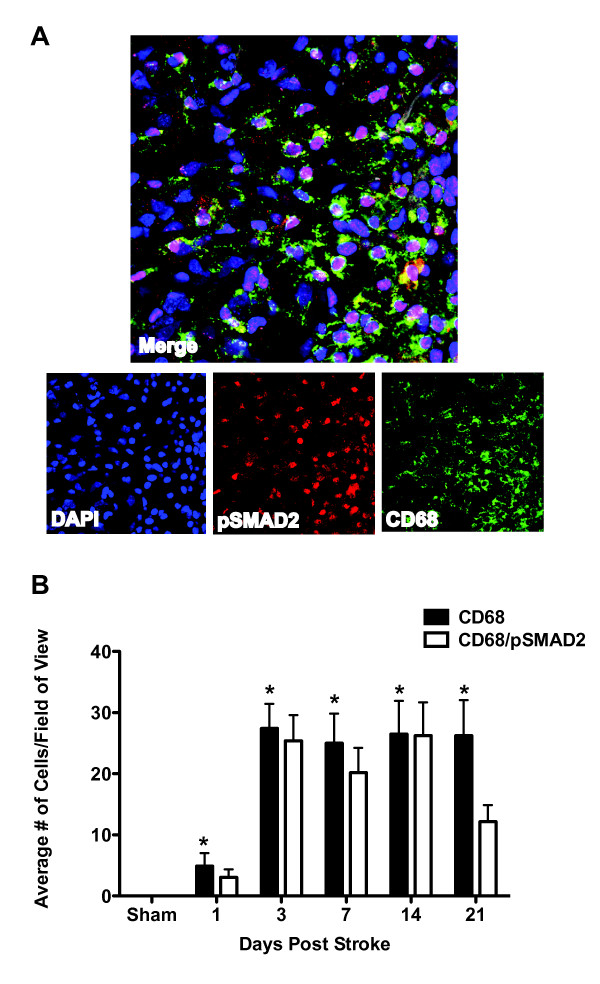
**TGFβ signaling in activated microglia/macrophages increases in the stroke penumbra after stroke**. A.) Representative image (40×) showing CD68+ activated microglia and macrophages) co-localized with pSmad2 after stroke. Image is from a young female mouse at day 3 post stroke. See additional file 5 for a 3D projection. B.) CD68 immunoreactivity increases in the first 3 days after stroke with the majority of CD68+ cells co-localizing with pSmad2 (*p < 0.05 compared to sham).

Although we found no difference in TGFβ signaling in neurons, oligodendrocytes and endothelial cells after stroke, we did find a qualitative increase in TGFβ signaling in astrocytes and microglia/macrophages. In the days following stroke, pSmad2 immunoflorescence became brighter in the border of the lesion (Fig 6A) compared to sham mice that showed even pSmad2 immunoflorescence throughout the cortex (Fig 6B). Concurrently, GFAP and CD68 immunoreactivity increased in the same location and the GFAP and CD68 positive cells co-localized intensely with pSmad2 (Fig 7 &8; see additional files 4 and 5 to confirm colocalization by 3D reconstructions). This was the case in young and old mice of each gender and increased co-localization of GFAP and CD68 with pSmad2 corresponded with the profile of increased TGFβ signaling after stroke we observed by real time imaging (Fig 2C-F). These data suggest that increased TGFβ signaling in the brain after stroke is due to responses from lesional and perilesional astrocytes and monocytic lineage immune cells.

## Discussion

We show here for the first time that TGFβ signaling in the brain increases in the first week after stroke in both young and old mice. We incorporated 18 month old mice into this study because nearly three quarters of all strokes occur in people over the age of 65 and there is a large knowledge gap regarding how mechanisms of recovery change with age. Similarly, we performed this study on mice of both genders in order to determine if there is a sex difference in TGFβ signaling after stroke. In 5 month old mice, TGFβ signaling increased 2 fold over baseline in the first week after stroke, before beginning to return to pre-stroke levels. This profile of increased TGFβ signaling was similar in 18 month old mice, although the absolute level of TGFβ signaling was significantly higher in the older animals. This could be in response to greater damage in the older animals as we found that lesion size was over double the volume compared to the younger mice. Baseline TGFβ signaling was also higher in the aged animals and so increased TGFβ signaling after stroke may also be a reflection of their higher baseline level. Our finding that infarct volume is increased in older animals conflicts with other studies that report that infarct volume is not increased in older subjects [18,20]. However, these studies used the suture model of stroke, which causes a larger lesion than the dMCAO model of stroke used here. Studies that use a similar cortical model of stroke to the dMCAO model find that lesion size is increased in aged rats relative to young [19]. Therefore the impact of age on lesion size appears to be stroke model dependent.

Every cell type in the brain has been shown to be capable of making TGF-β1 and increases in TGF-β1 mRNA have been demonstrated after stroke [1,21]. To discover which cell type is predominantly responsible for post stroke production of TGF-β1 we co-localized an anti-TGF-β1 antibody with markers of different brain cell types. We found that TGF-β1 predominantly co-localized with CD68, a marker of activated microglia and macrophages, indicating that they are likely to be the cells that produce it after stroke.

While there is ample evidence that TGFβ production is increased after stroke, as it is after many kinds of brain injury, it was not known whether increased TGFβ production translates to an increased response, because all TGFβ isoforms are excreted as an inactive form that lies inert in the extracellular matrix [22]. TGFβ receptor insertion into the membrane is highly regulated and the intracellular Smad pathways that transmit TGFβ signals interact with a complex array of other kinase substrates [23]. Therefore, although many of the proteins that activate TGFβs are upregulated after brain injury, such as reactive oxygen species, metalloproteases, plasmin, and thrombospondin [22], it was not known if increases in TGF-β1 mRNA would correlate directly with increased TGFβ signaling after stroke. And in fact we found that TGFβ signaling was regulated differently in different cell types after stroke.

TGF-β1 is strongly neuroprotective, at least in part due to direct effects in neurons [11,24-28]. It is also involved in synapse formation, the balance of excitatory and inhibitory transmission in the hippocampus and plasticity in multiple circuits [29]. We found that TGFβ signaling in neurons is widespread and consistent before stroke and that there are no obvious quantitative or qualitative differences after stroke. This suggests that while TGFβ signaling plays an important constitutive function in neurons, the physiological role of increased TGF-β1 after stroke may not be to signal to neurons.

Surprisingly, we also found that the majority of oligodendrocytes in the brain are responding to TGFβ in the absence of injury and after stroke. This suggests that TGFβ signaling plays a physiological role in oligodendrocytes that was heretofore unappreciated. As with neurons, co-localization of pSmad2 with oligodendrocytes did not increase after stroke suggesting that the role of increased TGF-β1 after stroke is not to communicate to this cell type. Whether constitutive TGFβ signaling in oligodendrocytes is important for their function or survival remains to be elucidated.

We rarely found co-localization of pSmad2 with endothelial cells. Signal transduction by TGFβ family members is mediated via specific heteromeric complexes of type I and type II serine/threonine kinase receptors. In most cells TGFβ signals via the type I receptor ALK5 but in endothelial cells it can also signal via the type I receptor ALK1 [7,30]. ALK5 induces phosphorylation of Smads 2 and 3 and ALK1 mediates phosphorylation of Smads 1, 5 and 8. Endothelial cells may not co-localize with pSmad2 due to the activation of the ALK1 pathway. Thus endothelial cells may still respond to TGFβ in the brain at baseline and after stroke but by activation of the Smads downstream of ALK1.

Our data demonstrates that CD68+ activated microglia and macrophages respond in an autocrine manner to the TGF-β1 they produce. We found that co-localization of CD68 with pSmad2 increased in parallel with TGFβ signaling reporter gene expression after stroke. TGFβ can exert either anti-inflammatory or pro-inflammatory effects in a context-dependent fashion. However, in the majority of contexts TGF-β1 appears to play an anti-inflammatory role and moderate the production of neurotoxic pro-inflammatory cytokines. It drives the differentiation of regulatory T cells and M2c macrophages that resolve immune responses, and resolves the inflammatory process in myocardial ischemia [7,31,32]. A function of TGF-β1 after stroke may be to drive the differentiation of monocytic lineage cells - both activated microglia and macrophages - into an M2c phenotype to enable a similar wound healing response to that identified in myocardial ischemia.

Our data also demonstrates that co-localization of pSmad2 with activated astrocytes increases after stroke. There is strong *in vitro *and *in vivo *evidence that TGFβ plays an important role in generating reactive astrogliosis, and that reactive astrocytes participate in innate immune responses. Overexpression of TGFβ chronically leads to reactive astrogliosis, and these mice display exacerbated reactive astrogliosis in a stab wound model [33]. Injection of TGF-β1 also increases reactive astrogliosis after stab wound [34], while injection of neutralizing antibodies inhibits expression of fibrogenic proteins, including proteoglycans, and reduces reactive astrogliosis [5,34]. Finally, Smad3 knockout mice display faster wound healing and decreased scar formation after brain stab injury [6]. Prior to this study no investigation addressed whether it is TGFβ signaling in astrocytes that leads to reactive astrogliosis, but we show here that astrocytes do respond to post stroke increases in TGFβ. Together this supports the hypothesis that TGFβ signaling in astrocytes directly mediates astrogliosis after stroke. Astrogliosis may have a detrimental effect on recovery due to astrocytes extending processes in the infarct border to encompass the developing infarct, thereby inhibiting neuronal plasticity and the formation of new axons and blood vessels in the infarcted region [35].

As the brain ages it becomes characterized by a low grade chronic pro-inflammatory state and responds with an earlier and stronger innate immune response following stroke [20]. We found that there is increased TGFβ signaling in the aged brain in the absence of injury and this may be part of an anti-inflammatory strategy employed by the aging brain to counter the primed pro-inflammatory state. Our findings are consistent with others who have shown that in aging the expression of TGFβ receptors increases, as does the expression of TGF-β1 [2,20,36,37]. Conversely, since the data we present here strongly suggests that TGFβ signaling is important for neuronal function, increased TGFβ signaling in the aged brain may be required to maintain normal neuronal function in the face of age related changes. More research is needed to determine which of these scenarios is correct.

## Conclusions

These results show that TGFβ signaling in the brain does not oscillate with the estrus cycle and that it increases 2 fold after the dMCAO model of stroke in young and old animals of each gender. Astrocytes and CD68+ innate immune cells are the cell types that respond to increased levels of TGFβ after stroke and they do so during the first week after stroke, coincident with glial scar formation. Given TGFβs effects on monocytic lineage cells and astrocytes we hypothesize that the role of increased TGFβ signaling after stroke is to regulate glial scar formation and the polarization of invading innate immune cells and resident microglia.

## Competing interests

The authors declare that they have no competing interests.

## Authors' contributions

KPD performed the surgery, estrus determination and live imaging. KPD, EC and LEM carried out the immunoflorescence. KPD and MSB conceived of the study, and participated in its design and coordination and helped to draft the manuscript. All authors read and approved the final manuscript.

## Supplementary Material

Additional file 1 **3D projection of neurons colocalizing with pSmad2**. 3D projection of Figure 4A. Click here for file

Additional file 2 **3D projection of oligodendrocytes colocalizing with pSmad2**. 3D projection of Figure 4B. Click here for file

Additional file 3 **3D projection of lack of colocalization of endothelial cells with pSmad2**. 3D projection of Figure 4C. Click here for file

Additional file 4 **3D projection of astrocytes colocalizing with pSmad2**. 3D projection of Figure 7A. Click here for file

Additional file 5 **3D projection of microglia colocalizing with pSmad2**. 3D projection of Figure 8A. Click here for file
